# Chondroitin sulfate disaccharide is a specific and sensitive biomarker for mucopolysaccharidosis type IVA

**DOI:** 10.1002/jmd2.12132

**Published:** 2020-06-30

**Authors:** Sharon J. Chin, Jennifer T. Saville, Belinda K. McDermott, Andreas Zankl, Janice M. Fletcher, Maria Fuller

**Affiliations:** ^1^ Genetics and Molecular Pathology SA Pathology [at Women's and Children's Hospital] Adelaide South Australia Australia; ^2^ Department of Clinical Genetics, The Children's Hospital at Westmead and Sydney Medical School The University of Sydney Sydney New South Wales Australia; ^3^ School of Medicine University of Adelaide Adelaide South Australia Australia

**Keywords:** biomarker, chondroitin sulfate, glycosaminoglycan, lysosomal storage disorder, Morquio syndrome, mucopolysaccharidosis

## Abstract

Mucopolysaccharidosis type IVA (MPS IVA) is an inborn error of glycosaminoglycan (GAG) catabolism characterized by a deficiency of the lysosomal enzyme, *N*‐acetylgalactosamine 6‐sulphatase (GALNS). Consequently, partially degraded GAG, chondroitin 6‐sulfate (CS) and keratan sulfate (KS), accumulate in the lysosomes of affected cells, primarily in cartilage resulting in skeletal disease. Excessive urinary excretion of these GAG is often used as the initial biochemical parameter to inform a laboratory diagnosis. Here we present the utility of a CS‐disaccharide with a non‐reducing 6‐sulfated *N*‐acetylgalactosamine residue (HNAc‐UA (1S))—the enzyme's substrate—for the diagnosis and biochemical monitoring of MPS IVA patients. Following implementation of this method into the diagnostic laboratory, we identified one MPS IVA patient over 3 years of MPS urine screening, with no false positive results from 2050 urines tested. Uniquely, urinary concentrations of HNAc‐UA (1S) are independent of age meaning that age‐related reference ranges are not required. Urinary HNAc‐UA (1S) was also able to identify two MPS IVA siblings who remained misdiagnosed with spondyloepiphyseal dysplasia for 5 years because of normal urinary GAG. HNAc‐UA (1S) could also be used as a biomarker for monitoring response to enzyme replacement therapy (ERT) as there was a drop in urinary concentration following the administration of ERT in all 12 patients and concentrations correlated with urinary KS (*R* ^2^ = 0.92). In conclusion, HNAc‐UA (1S) is a reliable, sensitive and specific biomarker for the diagnosis of MPS IVA and can be used to biochemically monitor the response to ERT.

**SYNOPSIS**

Urinary measurement of a disaccharide derived from chondroitin sulfate can be used for the diagnosis of MPS IVA independent of age and is useful to monitor the pharmacodynamic efficacy of enzyme replacement therapy correlating with keratan sulfate.

## 1 INTRODUCTION

Mucopolysaccharidosis type IVA (MPS IVA, Morquio syndrome) is one of the 11 mucopolysaccharidoses arising due to inherited deficiencies in lysosomal enzymes required for the degradation of glycosaminoglycans (GAG). The biochemical hallmark is lysosomal accumulation of incompletely digested GAG, which leads to the progressive deterioration of cells, tissues and organs.^1^ In MPS IVA the deficient enzyme, *N*‐acetylgalactosamine 6‐sulphatase (GALNS), is responsible for hydrolysing *N*‐acetyl‐D‐galactosamine 6‐sulfate residues within chondroitin 6‐sulfate (CS), as well as de‐sulphating galactose 6‐sulfate residues present in keratan sulfate (KS).^2^ Given CS and KS are primarily distributed in the cartilage, impaired degradation and subsequent accumulation of this GAG manifests primarily as deformities of the skeleton including short stature and joint laxity; cardiopulmonary and visual impairments are also often apparent.^3^

The diagnosis of MPS IVA can be challenging, compared with other MPS types. This is because initial laboratory investigations for MPS most commonly use a simple dye binding assay that measures total urinary GAG excretion, notably elevated in the MPS.^4^, ^5^ However, in the case of MPS IVA this test can miss the presence of urinary KS because the total amount of GAG may not be elevated beyond the normal range, thereby producing false negative results.^6^, ^7^, ^8^ Qualitative analyses via electrophoresis or thin layer chromatography attempts to identify the type of GAG present in the urine and typically identifies the KS in MPS IVA urine, but this secondary testing is usually only reflexed after raised total GAG is reported in the preliminary urine screen.^6^, ^9^

The introduction of mass spectrometric approaches into the diagnostic testing arena has improved the sensitivity and specificity for MPS compared with colourimetric and electrophoretic techniques.^10^, ^11^ For MPS IVA, the sensitivity of KS detection in urine have been achieved by enzymatic depolymerization of the polysaccharide with keratanase generating disaccharides that can be detected by mass spectrometry.^12^ With the availability of enzyme replacement therapy (ERT) for MPS IVA, early and accurate diagnosis is paramount to enable patients to commence specific treatment. We have previously reported a simple spot urine test that quantifies a CS‐disaccharide with a terminal, nonreducing *N*‐acetylgalactosamine 6‐sulfate residue (HNAc‐UA (1S))—the substrate for the enzyme (GALNS)—that does not rely on deconstruction of the polysaccharides. This approach is based on the premise that each lysosomal enzyme involved in the degradation of GAG, exclusively acts on the nonreducing end such that the incompletely degraded GAG that accumulate in each MPS type reflect the substrate specificity of the enzyme deficiency. Therefore, HNAc‐UA (1S) is unique to MPS IVA and shows absolute specificity for MPS IVA making it a useful and practical diagnostic biomarker.^11^ Here we report on the utility of this CS‐disaccharide (HNAc‐UA (1S)) in the laboratory setting with the prospective diagnosis of one MPS IVA patient since implementing this test 3 years ago, and importantly, demonstrate the identification of two siblings with MPS IVA who were missed with traditional urine GAG testing but returned elevated urinary concentrations of HNAc‐UA (1S). Additionally, we show that measurement of this HNAc‐UA (1S) can be used as a pharmacodynamic biomarker for biochemical monitoring the response to ERT and correlating with urinary KS.

## 2 MATERIALS AND METHODS

### 2.1 Patient samples

Urine samples were submitted to our Department for Diagnosis or Biochemical Monitoring of MPS and the Institutional Human Ethics Committee approved the use of these samples in this study (HREC/15/WCHN/69). Clinical information was available for two siblings, one sibling (S1) presented at 7 years of age with symptoms of knee pain and sibling two (S2) presented at 6 years of age with short stature.

### 2.2 Urinary GAG, KS, and one dimensional high resolution electrophoresis

Total urinary GAG was measured using the dimethylmethylene blue (DMB) method.^5^ Urinary KS was quantified at ARUP Laboratories^12^ and one dimensional high resolution electrophoresis (HRE) was performed as detailed previously.^13^

### 2.3 CS‐disaccharide determination

The CS‐disaccharide (HNAc‐UA (1S)) was quantified in spot urine samples by liquid chromatography electrospray ionization‐tandem mass spectrometry as described previously.^11^ High and low quality control (QC) material were prepared from urine of a previously diagnosed MPS IVA patient using 0.5 and 0.25 μmole of creatinine equivalents, respectively.

### 2.4 Enzyme activities

Leukocytes were resuspended in 0.1% Triton X‐100 and lysed by five cycles of freeze‐thaw. The protein concentration of the resultant homogenate was determined by the method of Lowry et al^14^ GALNS activity was measured using the protocol provided by the substrate vendor (Moscerdam Substrates, Oegstgeest, Netherlands; Protocol M4A, March 2013) and based on that of van Diggelen et al^15^ Arylsulphatase A activity was measured by the method of Baum et al.^16^ using 0.1 mL of leukocyte homogenate.

### 2.5 Molecular analysis of the *GALNS* gene

Next generation sequencing was performed on an Illumina HiSeq and analysis was restricted to the coding regions and canonical splice sites of the *GALNS* gene. Alignments and variant calls were generated using SoftGenetics NextGene with an allele frequency of <1%, and variant classification was in accordance with the American College of Medical Genetics (ACMG) criteria.^17^

## 3 RESULTS

### 3.1 Prospective diagnosis of MPS IVA

The CS‐disaccharide (HNAc‐UA (1S)) has previously been shown to be diagnostic for MPS IVA.^11^ Since implementation of this assay within the diagnostic laboratory we have identified one MPS IVA patient with elevated concentrations in urine (0.41 mmol/mol creatinine; RR < 0.1). Leukocyte GALNS activity in this patient was significantly reduced at 0.1 nmol/17 hours/mg protein (RR 90‐360 nmol/17 hours/mg protein) and molecular testing confirmed this patient to be homozygous for the c.433C>T (p.His145Tyr) pathogenic variant. This patient was identified from a total of 2050 MPS tests performed during the last 3 years with no false positive results and no reported false negative results. Additionally, during this time, proficiency testing as assessed by ERNDIM correctly identified 2/2 MPS IVA samples.

### 3.2 Diagnosis of MPS IVA in siblings S1 and S2

**FIGURE 1:**
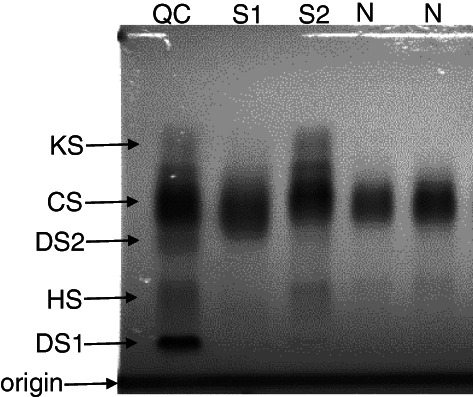
High resolution electrophoresis of urinary GAG. QC, quality control material containing a mixture of MPS type II and type IV urine to generate two dermatan sulfate bands (DS 1 and 2), heparan sulfate (HS), chondroitin 6‐sulfate (CS) and keratan sulfate (KS) as described in Hopwood and Harrison (1982). S1 and S2 denotes urine from sibling 1 and 2, respectively and N denotes two urine samples from unaffected individuals

Radiographs from two siblings showed mild features of dysostosis multiplex suggestive of MPS (Figure S1). Initial laboratory investigations on these two siblings were normal (Table 1) and as such were considered to exclude a laboratory diagnosis of MPS. The siblings were diagnosed with spondyloepiphyseal dysplasia (SED) of an unknown subtype. This diagnosis of SED remained for 5 years, however, persistent clinical suspicion of MPS IVA led to GALNS determinations in leukocytes from both siblings, which returned enzyme activity significantly below the normal range (Table 1). Analysis of a second sulphatase, arylsulphatase A, excluded the possibility of multiple sulphatase deficiency and confirmed the viability of the leukocyte preparation. Confirmatory molecular testing found the siblings to be heterozygous for two previously reported pathogenic variants c.143 T>G (p.Val48Gly) and c.331C>T (p.Gln111*). Of note, the siblings repeat urinary total GAG and HRE pattern were incongruent with one sibling returning elevated total urinary GAG and a normal HRE pattern and the second, normal total urinary GAG with increased KS on HRE (Table 1 and Figure 1). These two urine samples were included in the deidentified cohort of samples for MPS testing^11^ and both were correctly identified as MPS IVA from controls and all other MPS subtypes based on elevated urinary HNAc‐UA (1S) (Table 1).

**TABLE 1:** Laboratory findings for S1 and S2

Biochemical parameter	Reference range	S1	S2
Total urinary GAG^a^ (g/mol creatinine)	<6.0	11.0	6.0
One dimensional GAG HRE^a^	N/A	normal	normal
Leukocyte GALNS activity (nmol/17 hours/mg protein)	90–360	14	9.6
Leukocyte arylsulphatase A (nmol/min/mg protein)	1.0‐5.0	1.5	1.4
Total urinary GAG (g/mol creatinine)	<6.0	8.0	4.0
One dimensional GAG HRE	N/A	normal	↑ KS
HNAc‐UA (1S) (mmol/mol creatinine)	<0.1	0.24	0.28

Abbreviations: GAG, glycosaminoglycan; GALNS, *N*‐acetylgalactosamine6‐sulphatase; HRE, high resolution electrophoresis; KS, keratan sulfate; N/A, not applicable.

a Initial laboratory findings at first presentation.

### 3.3 Biochemical monitoring

**FIGURE 2:**
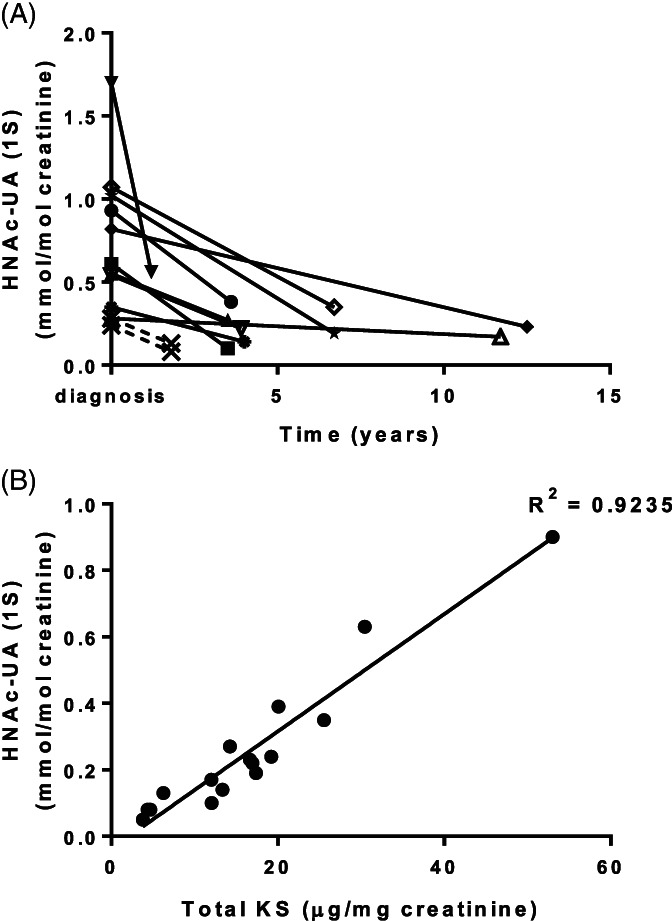
CS‐disaccharide(HNAc‐US (1S)) in urine of MPS IVA patients. A, Urinary HNAc‐UA (1S) concentrations in 12 patients at diagnosis and following enzyme replacement therapy (ERT) ● 5.8 years at diagnosis, 3.6 years posttreatment (59% drop); ▪ 10.9 years at diagnosis, 3.5 years posttreatment (84% drop); ▴ 2.1 years at diagnosis; 3.5 years posttreatment (51% drop); ▼ 1.4 years at diagnosis, 1.2 years posttreatment (67% drop); ◆ 1.9 years at diagnosis, 12.5 years posttreatment (72% drop); **X** S1 and S2, 12.7 and 11.7 years at diagnosis, respectively, both 1.8 years posttreatment (67% and 54% drop, respectively); △ 3.6 years at diagnosis, 11.7 years posttreatment (39% drop); ▽ 5.1 years at diagnosis, 3.9 years posttreatment (59% drop); ▽ 1.5 years at diagnosis, 6.7 years posttreatment (67% drop);* 4.6 years at diagnosis, 4 years posttreatment (60% drop); ⋆ 1.5 years at diagnosis, 6.7 years posttreatment (81% drop). B, Correlation between HNAc‐UA (1S) and total KS in 16 archived MPS IVA urine samples post‐ERT

For biochemical monitoring of patients receiving ERT it was necessary to demonstrate linearity of HNAc‐UA (1S) to afford accurate quantification. Dilution of MPS IVA patient urine with control urine revealed linearity over the biological range of 0.01‐5HNAc‐UA (1S) mmol/mol creatinine (Figure S2). Interassay variation was calculated at 20% for both the low and high QC over 17 separate runs. Spot urine samples were available from 12 patients pre‐ and post‐ERT, including samples for S1 and S2, with all patients showing a reduction in HNAc‐UA (1S) posttreatment (Figure 2A). Additionally, from 16 archived MPS IVA urine samples post‐ERT, the concentration of urinary HNAc‐UA (1S) correlated with total KS concentrations (Figure 2B).

## 4 DISCUSSION

Variability in phenotypic expression of MPS often delays diagnosis and the case of two MPS IVA siblings described herein highlights the pitfalls in traditional laboratory methods routinely used for “ruling out” an MPS. We have previously shown that naturally occurring low molecular weight GAG fragments with nonreducing end residues defined by the nature of the enzyme deficiency are specific for each MPS subtype enabling accurate diagnosis of MPS.^11^ The particular structure for MPS IVA is a disaccharide, HNAc‐UA (1S), which presumably contains a 6‐sulfated *N*‐acetyl‐D‐galactosamine residue at the non‐reducing end ‐ the substrate for the defective enzyme. However, as the mass spectrometric detection method, which measures the disaccharide based on its mass‐to‐charge ratio, is unable to distinguish stereoisomers nor determine the position of the sulfate moiety, the structure of this disaccharide remains an assumption. This disaccharide is not known to be elevated in any other MPS type, and from 2050 cases with a clinical suspicion of MPS, one patient was correctly identified as MPS IVA with elevated HNAc‐UA (1S); no false positives and no recognized false negatives were identified throughout 3 years of testing. The accuracy of this method was further highlighted through external proficiency testing (ERNDIM) that correctly identified MPS IVA samples with no false positive or negative results. Testament to the diagnostic utility of HNAc‐UA (1S) was the identification of two MPS IVA siblings that were incorrectly diagnosed with SED after initial laboratory testing returned normal results (Table 1).

Although the measurement of urinary KS following depolymerization of the GAG polymer has provided a reliable laboratory test for MPS IVA,^12^ KS is elevated in other MPS types and unrelated conditions and is therefore not specific for MPS IVA.^7^ Additionally, as KS concentrations are highest when children are growing and thereafter as the growth plate starts to close concentrations decrease, urinary KS declines rapidly following adolescence.^12^ This can be problematic not only for diagnosis but also for longitudinal biochemical monitoring of patients following therapeutic intervention because KS will fluctuate with physiological growth and development.^18^ However, urinary HNAc‐UA (1S) levels are independent of age^11^; included in this control cohort were 48 urines from infants <1 month of age, 196 between 1 and 12 months, 769 between 1 and 5 years and 981 urines from individuals >5 years of age, all returning HNAc‐UA (1S) concentrations <0.1 mmol/mol creatinine. A precipitous drop in concentration was demonstrated following initiation of ERT in 12 MPS IVA patients compared with concentrations at diagnosis (Figure 2A) in all patients, regardless of age and time at follow‐up. The youngest patient was 1.4 years at the time of diagnosis and returned the highest concentration of HNAc‐UA (1S) at 1.7 mmol/mol creatinine, whereas the oldest patient at 12.7 years returned the lowest concentration at 0.24 mmol/mol creatinine suggesting a relationship between age of onset and the disaccharide. Although only 12 patients were included in this study there was a weak correlation between age at diagnosis and urinary HNAc‐UA (1S) concentration (Pearson's r = −0.5; *P* = .05). Larger cohort studies are required to determine whether this CS‐disaccharide is informative for assessing disease burden and predicting symptom onset in MPS IVA patients.

Recently, Lawrence et al.^19^ employed enzymatic depolymerization of the high molecular weight CS to generate the monosaccharide, *N*‐acetyl‐D‐galactosamine 6‐sulfate, which is the substrate for the deficient enzyme. Similar to our study, they reported that the monosaccharide was less affected by age than urinary KS determinations and demonstrated that it is a disease‐specific biomarker for MPS IVA. Although they also observed a reduction in this monosaccharide in response to ERT, differences were noted in some patients when compared to measuring total urinary KS. This may be due, at least in part, to the source of KS measured as circulating levels are related to proteoglycan turnover within cartilage whereas urinary KS is derived from the kidney either directly or passes through the glomerular filtrate. As only KS molecules small enough to transverse the glomerular capillary membrane will end up in the urine, urinary KS determinations will not include larger fragments. Therefore, fragments of CS may indeed present a better biomarker for substrate burden in MPS IVA than KS and the measurement of the CS‐disaccharide, which did correlate with urinary KS, may therefore better reflect CS storage than the monosaccharide, *N*‐acetyl‐D‐galactosamine6‐sulfate.

Further work is needed to explore the relationship between CS, KS and oligosaccharide fragments as disease biomarkers for MPS IVA. HNAc‐UA (1S) is a naturally produced disaccharide generated from the initial action of endo‐β‐*N*‐acetylhexosaminidases on the large polysaccharide chains of CS, producing oligosaccharides that are subsequently degraded from their non‐reducing termini by the action of specific lysosomal exoenzymes that reduce these oligosaccharides to monosaccharides and inorganic sulfate for lysosomal egress. In MPS IVA the absence of GALNS activity produces a series of oligosaccharides with terminal *N*‐acetyl‐D‐galactosamine 6‐sulfate residues.^20^, ^21^ Presumably the small size of HNAc‐UA (1S) (a disaccharide), allows it to be excreted in the urine, but its relationship to the overall GAG storage burden in MPS IVA and connection with CS accumulation in the skeletal system or otherwise remains unknown.

## 5 CONCLUSION

In conclusion, the CS‐disaccharide, HNAc‐UA (1S), is a specific and sensitive biomarker for MPS IVA that is easily measured in urine in a multiplex assay enabling detection of all MPS types. Importantly, age related reference ranges are not required as the concentrations of HNAc‐UA (1S) do not vary with physiological growth. HNAc‐UA (1S) can also be used to monitor the pharmacodynamic efficacy of ERT, analogous to urinary KS, but with the advantage that enzymatic or chemical depolymerization of the GAG is not required.

## CONFLICT OF INTEREST

Jennifer Saville, Sharon Chin, Belinda McDermott, and Andreas Zankl declare they have no conflict of interest. Janice Fletcher and Maria Fuller have received travel support for attendance at conferences and educational meetings from BioMarin.

## AUTHOR CONTRIBUTIONS

Jennifer T. Saville, Sharon J. Chin, Janice M. Fletcher and Maria Fuller designed the study. Jennifer T. Saville, Sharon J. Chin, and Belinda K. McDermott performed the experimentation. Andreas Zankl provided patient and clinical data. Jennifer T. Saville, Sharon J. Chin, and Maria Fuller analyzed the data and wrote the manuscript. All authors reviewed and intellectually contributed to the final version.

## ETHICS STATEMENT

The use of patient samples in this study was approved by the Women's and Children's Health Network Human Research Ethics Committee (HREC/15/WCHN/69).

## INFORMED CONSENT

All procedures followed were in accordance with the ethical standards of the responsible committee on human experimentation (institutional and national) and with the Helsinki Declaration of 1975, as revised in 2000. Individual patient consent was not sought as all procedures were in accordance with routine patient care.

## ANIMAL ETHICS APPROVAL

This article does not contain any studies with animal subjects performed by any of the authors.

## Supporting information


**Appendix S1**: Supplementary Material
