# Supplementary material for: Chondroitin sulfate disaccharide is a specific and sensitive biomarker for mucopolysaccharidosis type IVA
Source: JIMD Rep. 2020 Jun 30;55(1):68–74. doi: 10.1002/jmd2.12132 (PMC7463049; doi:10.1002/jmd2.12132)
Supplement: Supplementary file 1 — Appendix S1: Supplementary Material [file JMD2-55-68-s001.docx]

**SUPPLEMENTARY MATERIAL**

**A chondroitin sulphate disaccharide is a specific and sensitive biomarker for mucopolysaccharidosis type IVA**

### Sharon J. Chin^1a^, Jennifer T. Saville^1a^, Belinda K. McDermott^1^, Andreas Zankl^2^, Janice M. Fletcher^1,3^ and Maria Fuller^1,3*^

^a^These authors contributed equally

^1^Genetics and Molecular Pathology, SA Pathology [at Women’s and Children’s Hospital], 72 King William Road, North Adelaide, South Australia, 5006; ^2^Department of Clinical Genetics, The Children’s Hospital at Westmead and Sydney Medical School, The University of Sydney, NSW; ^3^School of Medicine, University of Adelaide, Adelaide, South Australia, 5005; Australia.

^*^Corresponding author:

Maria Fuller, Genetics and Molecular Pathology, SA Pathology [at Women’s and Children’s Hospital], 72 King William Road, North Adelaide, South Australia, 5006, Australia

email: maria.fuller@adelaide.edu.au.


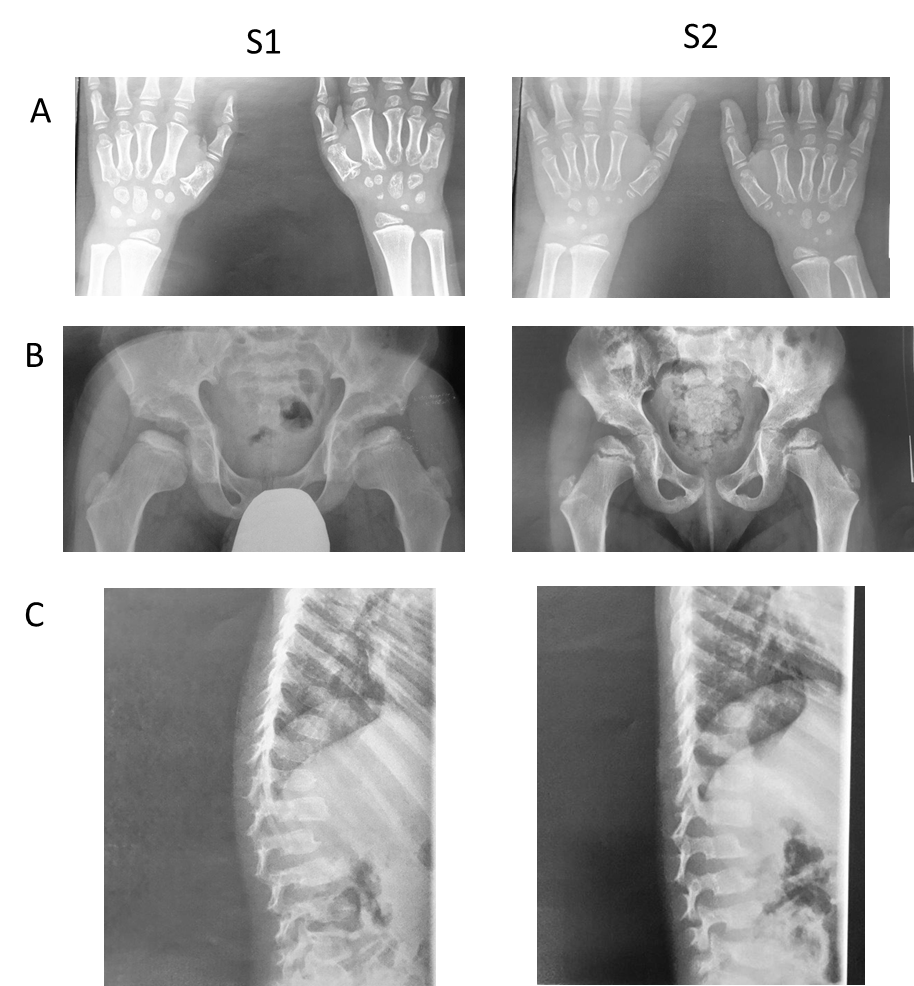


**SFig. 1.** Radiographs of sibling 1 (S1) and sibling 2 (S2). A. Hands showing short and broadened metacarpals with thin cortices and proximal pointing. B. Pelvis/hips with bilateral flattening of the capital femoral epiphyses and irregular acetabulae. C. Spine showing anterior beaking of the spinal body.

**Linearity of chondroitin sulphate (CS) disaccharide in urine**

To ensure the CS-disaccharide HNAc-UA (1S) was quantitative, a standard curve was prepared using urine from a known MPS IVA patient mixed with control urine to give a total of 0.5 µmoles creatinine as shown in Table S1. Combined samples were lyophilised prior to derivatisation and quantification by liquid chromatography-mass spectrometry as previously described.^11^ SFig. 2 shows that HNAc-UA (1S) was linear across the concentration range.

**Table S1**: Preparation of standard curve from MPS IVA patient urine mixed with control urine to a total of 0.5 µmoles of creatinine.

| creatinine (µmoles) | |
| --- | --- |
| MPS IVA | control |
| 0.5 | 0 |
| 0.4 | 0.1 |
| 0.3 | 0.2 |
| 0.2 | 0.3 |
| 0.1 | 0.4 |
| 0.05 | 0.45 |
| 0.01 | 0.49 |
| 0.005 | 0.495 |

**SFig. 2:** Linearity of HNAc-UA (1S) over the biological range.
